# Disrupted topological properties of the structural brain network in patients with cerebellar infarction on different sides are associated with cognitive impairment

**DOI:** 10.3389/fneur.2022.982630

**Published:** 2022-09-20

**Authors:** Duohao Wang, Qun Yao, Xingjian Lin, Jun Hu, Jingping Shi

**Affiliations:** ^1^Department of Neurology, Affiliated Brain Hospital of Nanjing Medical University, Nanjing, China; ^2^Department of Radiology, Affiliated Brain Hospital of Nanjing Medical University, Nanjing, China

**Keywords:** cerebellar infarction, cognitive impairment, brain network, diffusion tensor imaging, graph theory

## Abstract

**Purpose:**

To explore changes in the brain structural network in patients with cerebellar infarction on different sides and their correlations with changes in cognitive function.

**Methods:**

Nineteen patients with acute left posterior cerebellar infarction and 18 patients with acute right posterior cerebellar infarction seen from July 2016 to September 2019 in the Department of Neurology, Affiliated Brain Hospital of Nanjing Medical University, were selected. A total of 27 healthy controls matched for sex, age, and years of education were recruited. The subjects underwent head diffusion magnetic resonance imaging examination and neuropsychological cognitive scale evaluation, and we analyzed changes in brain structural network properties in patients with cerebellar infarction and their correlation with changes in patients' cognitive function.

**Results:**

The Mini-Mental Status Examination (MMSE), Montreal Cognitive Assessment (MOCA) and the Rey auditory verbal learning test (RAVLT) scores in the left and right cerebellar infarction groups were significantly lower than those in the healthy control group (*p* < 0.05). In addition, the digit span test (DST) scores were lower in the left cerebellar infarction group (*p* < 0.05); the trail-making test (TMT) times in the right cerebellar infarction group were significantly higher than those in the left cerebellar infarction group (*p* < 0.05). Meanwhile, the left and right cerebellar infarction groups had abnormal brain topological properties, including clustering coefficient, shortest path length, global efficiency, local efficiency and nodal efficiency. After unilateral cerebellar infarction, bilateral cerebral nodal efficiency was abnormal. Correlation analysis showed that there was a close correlation between decreased processing speed in patients with left cerebellar infarction and decreased efficiency of right cerebral nodes (*p* < 0.05), and there was a close relationship between executive dysfunction and decreased efficiency of left cerebral nodes in patients with right cerebellar infarction (*p* < 0.05).

**Conclusion:**

Patients with cerebellar infarction have cognitive impairment. Unilateral cerebellar infarction can reduce the network efficiency of key regions in the bilateral cerebral hemispheres, and these abnormal changes are closely related to patient cognitive impairment. The results of this study provide evidence for understanding the underlying neural mechanisms of cerebellar cognitive impairment and suggest that brain topological network properties may be markers of cerebellar cognitive impairment.

## Introduction

The role of the cerebellum in voluntary movement control, muscle tone regulation, and maintenance of body balance is widely known. In recent years, studies in neuroanatomy, physiology, behavior and other fields have confirmed that the cerebellum also has nonmotor functions, especially in the higher cognitive functions of humans (1–3). In 1998, Schmahmann proposed the concept of “cerebellar cognitive affective syndrome” (CCAS), which mainly manifests as visual-spatial impairment, executive dysfunction, language deficits, and affective disorders (4).

Recent studies have shown that the output of the cerebellum targets multiple nonmotor areas in the cerebral cortex as well as motor areas. Projections to different cortical regions originate from different output channels within the cerebellar nuclei. These cerebrocerebellar loops provide the cerebellum with the anatomical substrate to influence motor and cognitive control (5). Neuroimaging and neuropsychological data provide strong support for this idea. Different areas of the cerebellum have different functions, and the area associated with cognitive function is located in the posterior lobe of the cerebellum (1). Posterior lobe lesions will lead to cerebellar cognitive affective syndrome.

Diffusion imaging technology (DTI) can noninvasively display brain network connectivity and functional status without exogenous stimulation or tasks. It has been widely used in the study of neuropsychiatric diseases, such as Alzheimer's disease, multiple sclerosis, and schizophrenia (6–8). As mentioned earlier, the cerebellum may be involved in the coordination of cognitive functions through cerebellar fiber circuits. Therefore, DTI methods can be used to explore changes in the brain structural network after the destruction of the cerebellum. To the best of our knowledge, this is the first DTI study on cognitive impairment in patients with acute cerebellar infarction.

In this study, patients with acute posterior cerebellar infarction on different sides and healthy subjects were selected to study changes in the brain structural network and network properties in patients with cerebellar infarction and their correlations with changes in cognitive function; to explore altered characteristics of the brain network in patients with cerebellar infarctions on different sides and their cognitive function; and to identify characteristic imaging markers to evaluate the correlations of and mechanisms underlying cerebellar and cognitive function to better guide clinical practice.

## Materials and methods

### Subjects

This study recruited 37 patients with acute unilateral posterior cerebellar infarction diagnosed in the Department of Neurology, Brain Hospital Affiliated to Nanjing Medical University from July 2016 to September 2019, who were aged 50–75 years, had an education level of≥6 years and had a disease course of 4–14 days; these patients included 19 patients with acute left cerebellar infarction and 18 patients with acute right cerebellar infarction.

The exclusion criteria were as follows: (1) stroke lesions that involved brain areas other than the posterior cerebellar lobe, and its large lesions or severe symptoms; (2) a previous history of stroke, leukoencephalopathy, brain tumor, encephalitis, metabolic disorders and other dementia caused by degenerative diseases of the nervous system; (3) mental disorders and intellectual disabilities; (4) a history of alcohol and drug abuse; and (5) inability to complete the examination due to various symptoms. In addition, 27 healthy subjects matched for the age, sex, and educational level of the patients in the experimental group were recruited as healthy controls.

The current study was approved by the Ethics Committee of the Affiliated Brain Hospital of Nanjing Medical University. Each participant provided written informed consent.

### Neuropsychologic testing

All participants underwent a battery of neuropsychological tests, with assessments conducted by psychometricians and psychologists. General mental status was assessed using the Mini-Mental Status Examination (MMSE) (9) and Montreal Cognitive Assessment (MOCA) (10). Episodic memory was assessed by the Rey auditory verbal learning test (RAVLT) (11). Attentional function was assessed with the digit span test (DST) (12). Execution functions were evaluated with the trail-making test (TMT) (13). Language function was assessed using the Boston naming test (BNT) (14). Visual-spatial abilities were assessed by the clock drawing test (CDT) (15). To assess the emotional state of the patients, we used the Hamilton Depression Scale (HAMD) (16).

### MRI data acquisition

All subjects underwent MRI scans at the Brain Hospital Affiliated to Nanjing Medical University using a Siemens 3.0T scanner (Erlangen, Germany) with a standard quadrature head coil. During the scan, the subjects kept quiet, closed their eyes, did not move, and relaxed their limbs.

The high-resolution T1-weighted images were collected in a sagittal direction using a 3D-SPGR sequence. The T1-weighted imaging parameters were as follows: 176 sagittal slices; 1.0-mm slice thickness; TR = 1900 ms; TE = 2.48 ms; flip angle = 9°; and matrix = 512 × 512. Conventional T2-weighted MR images were obtained to rule out cortical atrophy and other brain abnormalities. Diffusion-weighted MR images were obtained using a spin echo planar imaging sequence, the parameters of which were as follows: TR = 6600 ms; TE = 93 ms; 45 axial slices; slice thickness = 3.0 mm; gap = 0 mm; 30 gradient directions with a *b* value = 1000 s/mm^2^; acquisition matrix = 128 × 128; FOV = 240 × 240 mm^2^.

Imaging was performed by physicians with over 10 years of experience in MR image acquisition.

### Data preprocessing

The data preprocessing process was completed with PANDA software and Gretna software. The specific steps included performing eddy current and head motion correction on DTI image data, removing excess scalp and brain tissue, and obtaining eigenvalues (λ1, λ2, λ3) and eigenvectors by diagonalizing 3 tensor matrices. Fractional anisotropy (FA) values were calculated. Simultaneously, matrices and inverses from a single space to a standard space were computed. The cerebral cortex and subcortical areas were divided into 90 functional areas using the automatic anatomical labeling (AAL) template, and each area was used as a network node (17). The FA image was used to construct the AAL standard image under a single spatial template, and the fiber network was constructed to generate 2 brains. A 90 × 90 matrix of mean FA values for all voxels along fiber bundles. Finally, a 3D visualization of the weighted network was constructed using BrainNet Viewer software. When building the brain network, we defined a threshold (*T* = 3) for determining the edges of the network; that is, if the number of connections between 2 nodes was greater than this threshold, it was considered that there was a connection between the 2 nodes (18).

### Topological properties

Graph theory is a theory used to describe the connection between two nodes, which is a graph composed of several nodes and edges. In brain networks, nodes and edges represent brain regions and connections between two regions, respectively. Brain networks have important topological parameters (19). In the current structural network study, we used several key parameters, including the clustering coefficient (Cp), normalized clustering coefficient (γ), shortest path length (Lp), normalized characteristic path length (λ), small-worldness (σ), local efficiency (Eloc), global efficiency (Eglob), and regional efficiency.

The clustering coefficient *C*(*i*) of node i represents the ratio of the actual number of edges to the number of fully connected edges of the subgraph. The clustering coefficient Cp of the network is defined as the average of the clustering coefficients of all nodes in the network, reflecting the connection state of the entire network. The formula is as follows:


$$\begin{array}{l} Cp\;=1/N\sum_{i=1}^{N}C\left(i\right). \end{array}$$

The shortest path length is indicated by *L*, and the shortest distance *Lij* from node *i* to node *j* refers to how many times the connection from node *i* can reach node *j*. The average of the distances of all nodes is the average shortest path length of the entire network. The shortest path length (Lp) measures the extent of network long-distance connections. The formula is as follows:


$$\begin{array}{l} Lp\left(G\right)=\frac{1}{N}\sum_{i\epsilon G}Li. \end{array}$$

Global efficiency (Eglob) measures the global efficiency of information transmission in the network. Global efficiency is defined as the average of the shortest path reciprocals of all nodes in the network. The formula is as follows:


$$\begin{array}{l} E_{\text{glob}}\left(G\right)=\frac{1}{N\left(N-1\right)}\sum_{i\neq j\epsilon G}1/Lij. \end{array}$$

Local efficiency (Eloc) is defined as the average of the inverse of the shortest path of all nodes in subgraph *Gi*. The formula is as follows:


$$\begin{array}{l} E_{\text{loc}}\left(G\right)=\frac{1}{N}\sum_{i\epsilon G}Eglob\;\left(Gi\right). \end{array}$$

Regional efficiency, Enodal(*i*), is a nodal property of structural networks, which indicates the average shortest path length between node *i* and all other nodes in the structural network. Enodal(*i*) is defined as follows:


$$\begin{array}{l} E_{\text{nodal}}\left(i\right)=\frac{1}{N-1}\underset{i\neq j\epsilon G}{\sum }1/Lij. \end{array}$$

Watts and Strogatz (20) refer to networks with both high clustering coefficients and shortest path lengths as small-world networks. If the network under study has a large clustering coefficient and approximate shortest path length relative to a random network, i.e., γ = Creal/Crandom >> 1, λ = Lreal/Lrandom ~ 1, then the network belongs to the category of a “small-world” network. Humphries et al. (21) unified the two metrics into a scalar σ = γ/λ to measure “small-world” properties; when σ > 1, the network has the “small-world” property.

### Statistical analysis

Statistical analysis was performed using SPSS 22.0 software. Age, educational level and neuropsychological test scores were compared using one-way ANOVA. The sex distribution was compared using the chi-square test. Network parameters were compared using one-way ANOVA followed by the LSD test for *post hoc* comparisons. False discovery rate correction was used to correct the statistical results of regional efficiency. Pearson correlation analyses assessed relationships between differences in brain structural network properties and neuropsychological test scores. Difference were considered statistically significant at *P* < 0.05.

## Results

### Comparison of clinical data and neuropsychological scores among the three groups of subjects

There were no significant differences in age, sex ratio, years of education, BNT score, HAMD score, or CDT score among the three groups (*p* > 0.05), and the MMSE, MoCA, and RAVLT scores between the left cerebellar infarction group and the right cerebellar infarction group were not statistically significant (*p* > 0.05). MMSE, MoCA, RAVLT, and DST scores in the left cerebellar infarction group and the right cerebellar infarction group were significantly lower than those in the HC group; TMT times were significantly higher, and the difference was statistically significant (*p* < 0.05). At the same time, the time taken to complete the TMT-B in the right cerebellar infarction group was significantly higher than that in the left cerebellar infarction group, and the difference was statistically significant (*p* < 0.05). See Table 1 for details.

**Table 1:** Demographic and clinical characteristics.

**Characteristic**	**Left cerebellar infarction (*n* = 19)**	**Right cerebellar infarction (*n* = 18)**	**Control subjects (*n* = 27)**	***F*-value**	***P*-value**
Age (year)	62.63 ± 7.30	59.78 ± 4.31	61.26 ± 4.73	1.24	0.29
Sex (M/F)	11/8	10/8	17/10	0.27	0.87^#^
Education (year)	8.16 ± 1.71	7.83 ± 0.99	8.41 ± 1.22	1.01	0.37
MMSE	26.68 ± 2.33	26.44 ± 1.89	27.81 ± 1.00	4.09	0.02^&^^$^
MOCA	22.37 ± 2.22	22.89 ± 2.76	27.04 ± 1.13	37.26	<0.001^&^^$^
RAVLT-A	21.16 ± 3.83	21.56 ± 3.26	23.78 ± 2.74	4.45	0.016^&^^$^
RAVLT-B	2.84 ± 1.34	3.0 ± 0.97	4.81 ± 1.0	23.03	<0.001^&^^$^
DST	10.68 ± 2.47	11.67 ± 2.45	12.44 ± 2.03	3.31	0.04^&^
BNT	24.74 ± 2.51	24.89 ± 3.10	26.19 ± 2.02	2.36	0.13
TMT-A	104.32 ± 46.06	104.56 ± 27.71	65.52 ± 12.66	13.01	<0.001^&^^$^
TMT-B	222.63 ± 37.09	257.72 ± 68.58	165.29 ± 50.39	17.47	<0.001^&^^#^^@^
HAMD	0.15 ± 0.50	0.22 ± 0.43	0.00 ± 0.00	2.38	0.101
CDT	3.84 ± 1.01	4.11 ± 0.90	4.48 ± 0.75	3.05	0.055

Values are represented as the mean ± SD;

# p-value for the sex distribution in the three groups was obtained using a χ^2^ test; other comparisons used the one-way analysis of variance (ANOVA); p < 0.05 was considered significant.

& *Post hoc* paired comparisons showed significant group differences between control subjects and patients with left cerebellar infarction.

$ *Post hoc* paired comparisons showed significant group differences between control subjects and patients with right cerebellar infarction.

@ *Post hoc* paired comparisons showed significant group differences between patients with left and right cerebellar infarction. MMSE, mini-mental state examination; MOCA, montreal cognitive scale; RAVLT, rey auditory verbal learning test; DST, digit span test; BNT, boston naming test; TMT, trail-making test; HAMD, Hamilton depression scale; CDT, clock drawing test.

#### Comparison of network properties among the three groups of subjects

**Figure 1:**
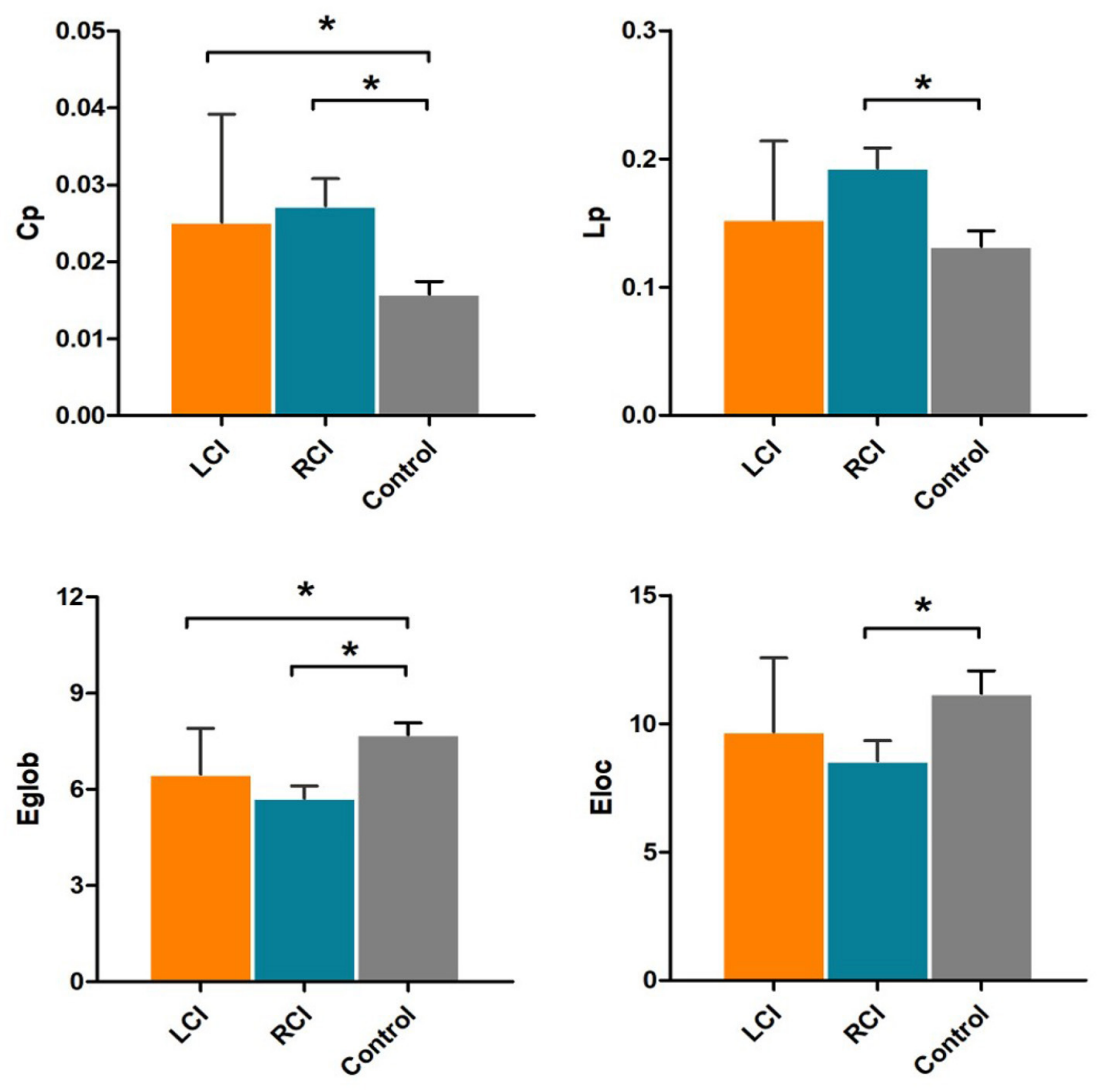
Significantly different network properties between patients with left cerebellar infarction, patients with right cerebellar infarction, and healthy controls. Bars and error bars indicate the means and standard deviations. The asterisk marks indicate significant differences between groups (*p* < 0.05). LCI, left cerebellar infarction; RCI, right cerebellar infarction.

There were statistically significant differences in the network properties CP, Lp, Eg and El among the three groups of subjects. The Cp value was significantly higher and the Eg value was significantly lower in the left cerebellar infarction group than in the healthy control group, and these differences were statistically significant (*p* < 0.05). The Cp value and Lp value were significantly higher, while the Eg value and the El value were significantly lower in the right cerebellar infarction group than in the healthy control group, and these differences were statistically significant (*p* < 0.05). There were no significant differences in network properties between the left cerebellar infarction group and the right cerebellar infarction group. Details are described in Figure 1.

### Comparison of regional efficiency among the three groups of subjects

**Figure 2:**
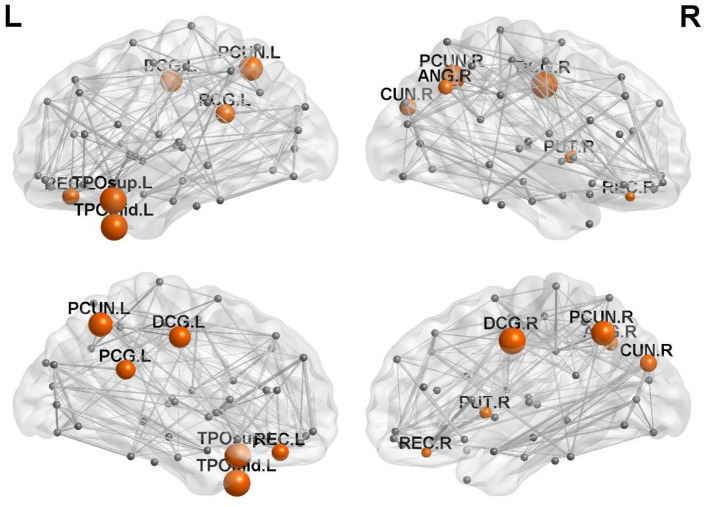
Comparison of nodal efficiency between the left cerebellar infarction group and the healthy control group; 12 brain regions with significant differences (*p* < 0.05, corrected for false discovery rate) between the two groups are shown in red. Node size represents the significance of group differences in nodal efficiency. PCUN, precuneus; DCG, median cingulate and paracingulate gyri; TPOmid, temporal pole, middle temporal gyrus; PUT, lenticular nucleus, putamen; REC, gyrus rectus; CUN, cuneus; ANG, angular gyrus; PCG, posterior cingulate gyrus; L, left; R, right.

**Figure 3:**
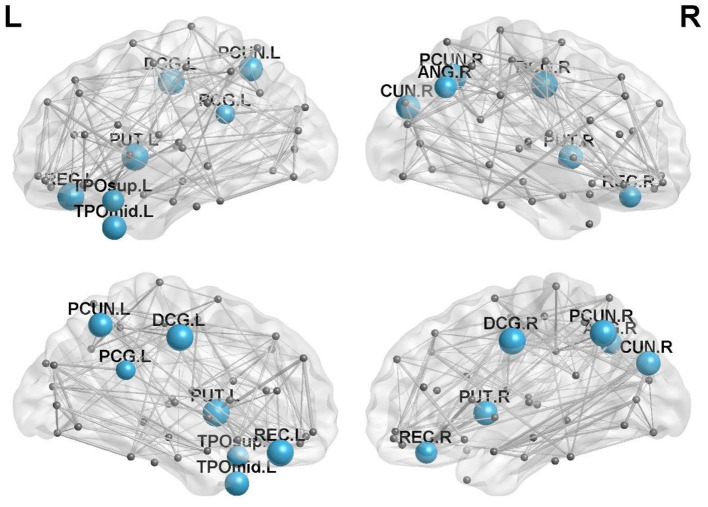
Comparison of nodal efficiency between the right cerebellar infarction group and the healthy control group; 13 brain regions with significant differences (*p* < 0.05, corrected for false discovery rate) between the two groups are shown in blue. Node size represents the significance of group differences in nodal efficiency. PCUN, precuneus; DCG, median cingulate and paracingulate gyri; TPOmid, temporal pole, middle temporal gyrus; PUT, lenticular nucleus, putamen; REC, gyrus rectus; CUN, cuneus. ANG, angular gyrus; PCG, posterior cingulate gyrus; L, left; R, right.

There were 13 brain regions with significant differences in regional efficiency among the three groups, including the bilateral precuneus (PCUN); bilateral median cingulate and paracingulate gyri (DCG); left temporal pole, middle temporal gyrus (TPOmid.L); left temporal pole, superior temporal gyrus (TPOsup.L); bilateral lenticular nuclei, putamen (PUT); bilateral gyrus rectus (REC); right cuneus (CUN.R); right angular gyrus (ANG.R); and left posterior cingulate gyrus (PCG.L). Compared with healthy controls, patients with left cerebellar infarction had significantly decreased efficiency in the following brain regions: bilateral precuneus (PCUN); bilateral median cingulate and paracingulate gyri (DCG); left temporal pole, middle temporal gyrus (TPOmid.L); left temporal pole, superior temporal gyrus (TPOsup.L); right lenticular nucleus, putamen (PUT.R); bilateral gyrus rectus (REC); right cuneus (CUN.R); right angular gyrus (ANG.R); and left posterior cingulate gyrus (PCG.L). See Figure 2 for details. The following brain regions showed significantly decreased efficiency in the patients with right cerebellar infarction compared with the healthy controls: bilateral precuneus (PCUN); bilateral median cingulate and paracingulate gyri (DCG); left temporal pole, middle temporal gyrus (TPOmid.L); left temporal pole, superior temporal gyrus (TPOsup.L); bilateral lenticular nucleus, putamen (PUT); bilateral gyrus rectus (REC); right cuneus (CUN.R); right angular gyrus (ANG.R); and left posterior cingulate gyrus (PCG.L). See Figure 3 for details. Brain regional efficiency was not significantly different in patients with left and right cerebellar infarction. Details are described in Table 2.

**Table 2:** Brain regions with significant differences in nodal efficiency among the patients with left cerebellar infarction, patients with right cerebellar infarction and control subjects.

**Region**	***F*-value**	***P*-value**	***Post hoc*** **test**		
			**LCI vs. control**	**RCI vs. control**	**LCI vs. RCI**
PCUN.R	10.68	<0.001	−3.27 (0.002)	−3.78 (<0.001)	ND
DCG.R	10.62	<0.001	−3.22 (0.001)	−4.10 (<0.001)	ND
DCG.L	9.64	<0.001	−2.84 (0.003)	−4.02 (<0.001)	ND
TPOmid.L	8.898	<0.001	−3.54 (0.001)	−3.14 (0.001)	ND
TPOsup.L	8.25	0.001	−3.40 (0.001)	−2.96 (0.002)	ND
PUT.L	7.84	0.001	ND	−4.13 (<0.001)	ND
PCUN.L	7.56	0.001	−3.08 (0.002)	−3.18 (0.001)	ND
REC.L	7.48	0.001	−2.53 (0.014)	−3.96 (<0.001)	ND
CUN.R	7.18	0.002	−2.84 (0.014)	−3.34 (0.001)	ND
PUT.R	7.04	0.002	−2.48 (0.025)	−3.55 (0.001)	ND
ANG.R	5.85	0.005	−2.61 (0.022)	−2.98 (0.002)	ND
REC.R	5.70	0.005	−2.07 (0.033)	−3.21 (0.002)	ND
PCG.L	5.37	0.007	−2.45 (0.011)	−2.92 (0.006)	ND

Comparisons of regional efficiencies were performed between LCI, RCI, and healthy controls using one-way ANOVA. *Post hoc* pairwise comparisons were then performed using t-tests. For ANOVA, P < 0.05 (false discovery rate corrected) was considered a significant difference. For *post hoc* tests, the LSD test and P < 0.05 indicated a significant difference. ND, no significant difference; PCUN, precuneus; DCG, median cingulate and paracingulate gyri; TPOmid, temporal pole, middle temporal gyrus; PUT, lenticular nucleus, putamen; REC, gyrus rectus; CUN, cuneus; ANG, angular gyrus; PCG, posterior cingulate gyrus; L, left; R, right.

### Relationship between brain network characteristics and neuropsychological scores in patients with cerebellar infarction

**Figure 4:**
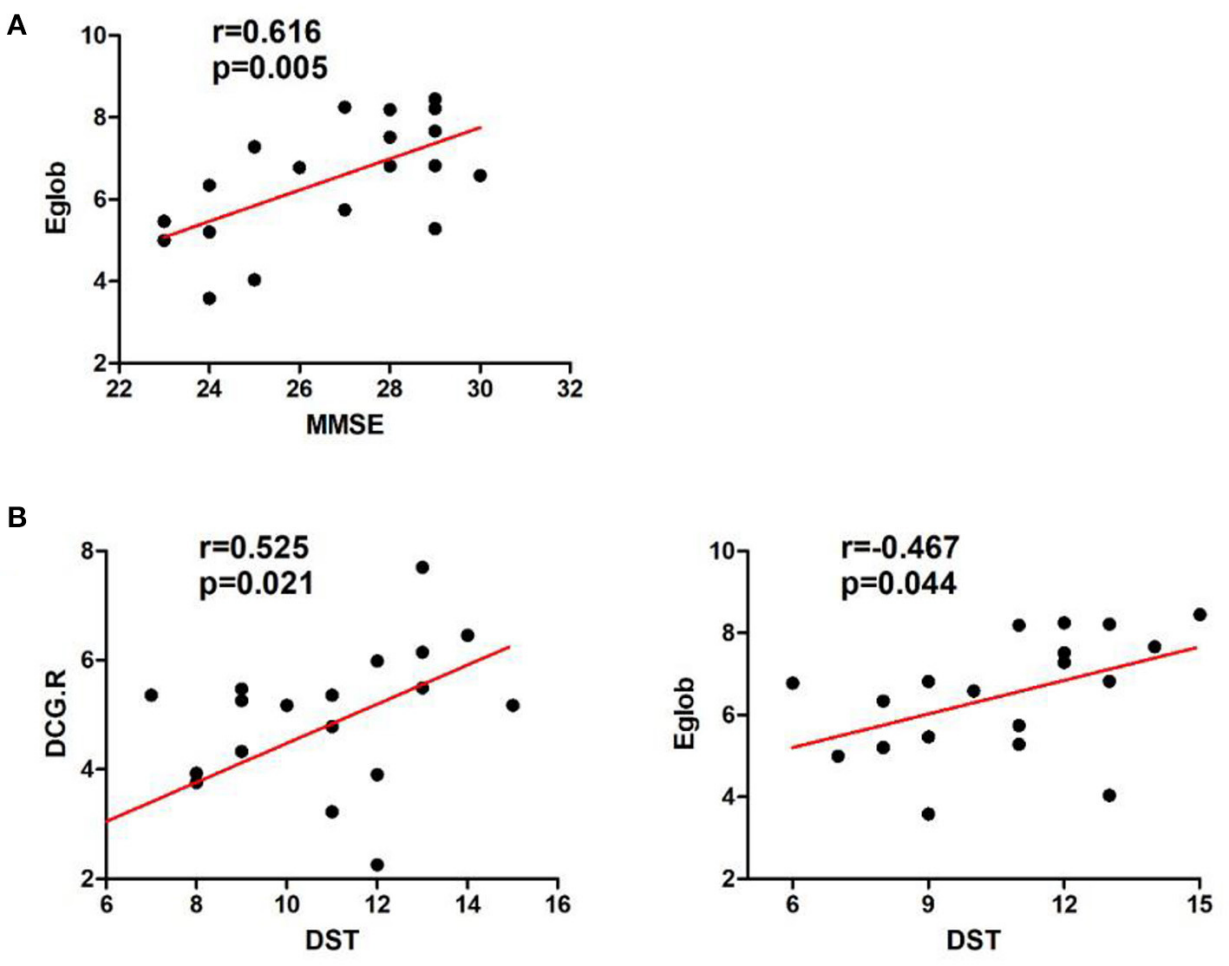
Correlations between network parameter values and neuropsychological scores in patients with left cerebellar infarction. **(A)** Eg values were positively correlated with MMSE scores. **(B)** Eg values and regional efficiency of the DCG.R were positively correlated with DST scores.

**Figure 5:**
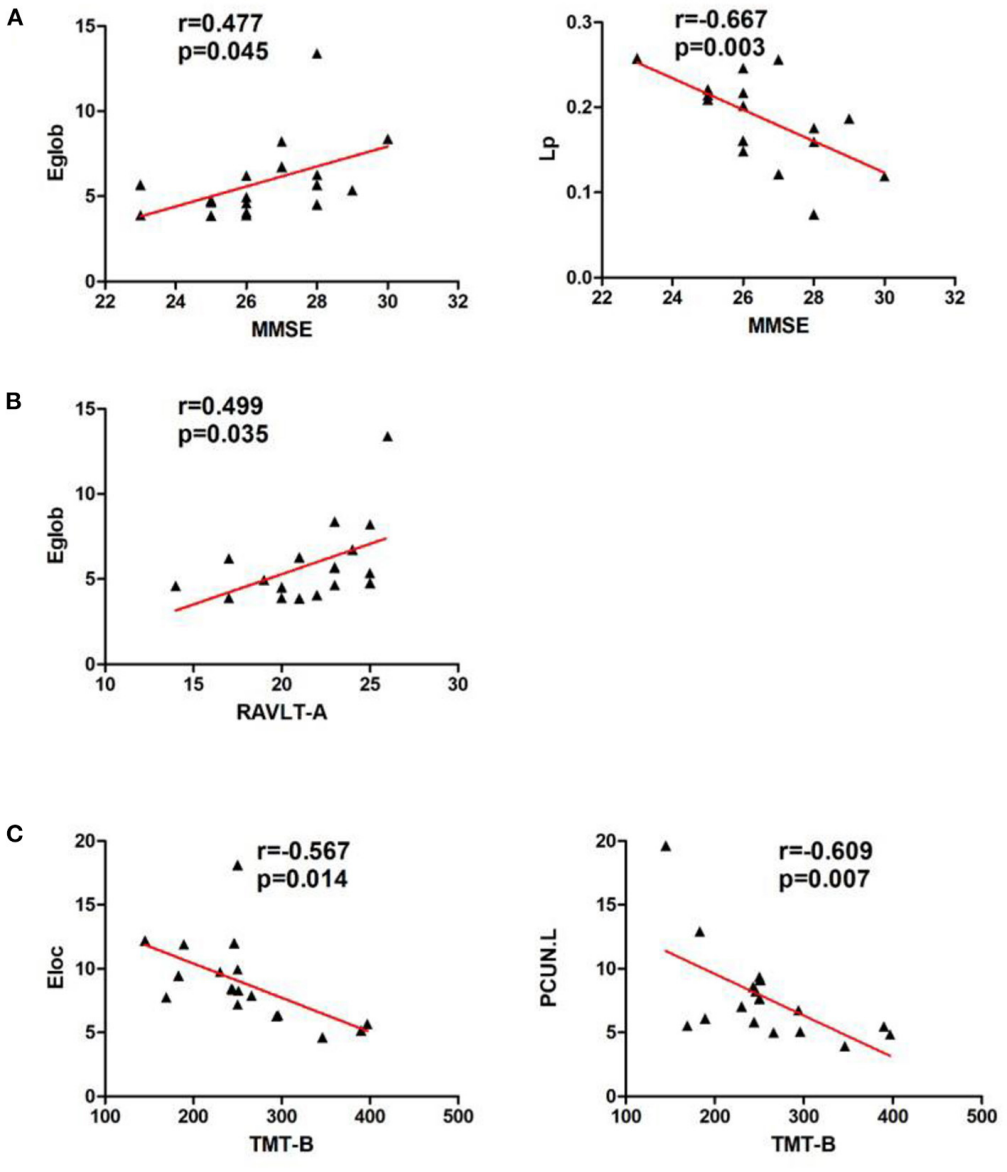
Correlations between network parameter values and neuropsychological scores in patients with right cerebellar infarction. **(A)** Lp values were negatively correlated with MMSE scores, and Eg values were positively correlated with MMSE scores. **(B)** Eg values were positively correlated with RAVLT-A scores. **(C)** El values and regional efficiency of the PCUN.L were negatively correlated with TMT-B scores.

In the left cerebellar infarction group, Eg values were positively correlated with MMSE scores (*p* < 0.05). In addition, Eg values and regional efficiency in the DCG.R were positively correlated with DST scores (*p* < 0.05). The details are shown in Figure 4. In the right cerebellar infarction group, Lp values were negatively correlated with MMSE scores (*p* < 0.05), and Eg values were positively correlated with MMSE and RAVLT-A scores (*p* < 0.05), El values and regional efficiency in the PCUN.L were negatively correlated with TMT-B scores (*p* < 0.05). Details are shown in Figure 5.

## Discussion

This study found that MMSE scores, MoCA scores, RAVLT scores and TMT scores of patients with cerebellar infarction, compared with healthy subjects, were significantly decreased, and the time spent on that TMT was significantly increased; furthermore, the time spent on the TMT in the right cerebellar infarction group was significantly higher than that in the left cerebellar infarction group. In addition, DST scores in the left cerebellar infarction group were significantly lower than those in the healthy control group. This suggests that there are changes in overall cognitive function (MMSE scores and MoCA scores), episodic memory (RAVLT scores) and executive function (TMT times) in patients with cerebellar infarction, and the degree of executive function impairment in the right cerebellar infarction group was more severe than that in the left cerebellar infarction group. Simultaneously, patients with left cerebellar infarction have impaired attentional function (i.e., DST scores). Several previous studies have shown that cerebellar lesions can lead to memory impairment, including episodic memory, attentional function, executive function, etc. (1, 22, 23). Some studies have also shown that the cerebellum may show laterality in cognition; that is, the left cerebellum is mainly related to visuospatial function (24), while the right cerebellum is mainly related to executive function (25).

In our study, we found that Cp values in the brain structural network in patients with left cerebellar infarction were significantly higher than those in healthy controls, and Eg values were significantly lower; in patients with right cerebellar infarction, Cp values and Lp values were significantly higher, and Eg values and El values were significantly lower. The lower Eg and El values indicated that the global efficiency and local efficiency of the brain structural network had decreased, suggesting that the brain structural network was disrupted after cerebellar infarction. Previous studies have shown that the cerebellum is involved in the regulation and coordination of motor and nonmotor functions through “cerebrocerebellar loops” (26). Previous studies have shown that the brain network has small-world properties; that is, it has a short Lp and a high Cp, which can ensure the efficient transmission of the brain network (21). In other words, increased Lp and decreased Cp indicate a decreased integration capacity of the brain network, i.e., disruption of the small-world properties (27). The increased Lp value of the brain network after right cerebellar infarction may indicate that there are potential changes in the small-world properties of the brain network after cerebellar lesions, which will lead to a decrease in the transmission efficiency of the brain network. On the other hand, we found that the Cp value of the brain network increased after left cerebellar infarction, which was inconsistent with the performance after the brain network was disrupted. We think this may represent a potential compensatory mechanism after disruption of the “cerebrocerebellar loops”, but we still need further research on this compensatory mechanism. In contrast, after right cerebellar infarction, there were no compensatory manifestations of increased brain network Cp values, and Lp values increased. Therefore, we believe that right cerebellar lesions may cause more severe brain structural network disruption than left cerebellar infarctions.

To clarify the functional changes in brain structural network nodes after bilateral cerebellar infarction, we investigated brain nodal efficiency. We found that there were 13 brain regions with decreased nodal efficiency after cerebellar infarction, including the bilateral precuneus; bilateral median cingulate and paracingulate gyri; left temporal pole, middle temporal gyrus; left temporal pole, superior temporal gyrus; bilateral lenticular nucleus, putamen; bilateral gyrus rectus; right cuneus; right angular gyrus; and left posterior cingulate gyrus. After unilateral cerebellar infarction, the effects were not simply limited to the ipsilateral or contralateral brain but appear to have effects on both sides of the brain. At the same time, we found that unilateral cerebellar infarction not only affects the ipsilateral or contralateral brain but also affects both sides of the brain. Cho et al. used repetitive transcranial magnetic stimulation (rTMS) of one side of the cerebellum and simultaneously performed 18 F-fluorodeoxyglucose and positron emission tomography to observe the metabolic changes in the brain. There was increased glucose metabolism in brain regions related to cognitive function, such as the left inferior frontal gyrus and the bilateral superior temporal gyrus (28). Therefore, we think unilateral cerebellar lesions may affect bilateral brain function.

In our study, we found that the efficiency of the precuneus, middle and posterior cingulate gyrus, and parts of the temporal lobe was reduced after cerebellar infarction. The default mode network is a hot spot in resting-state brain functional network research, and the network mainly includes the precuneus, posterior cingulate cortex, and bilateral temporal cortices (29). Cognitive impairment was shown to be closely related to disruption of the default network in multiple studies of brain function in Alzheimer's disease (30, 31). Available research has shown that the precuneus is involved in visuospatial processing, cognition, and consciousness, and there are functional connections between the precuneus and the frontotemporal and inferior parietal lobes (32, 33). A study of brain functional connectivity in obsessive-compulsive disorder found key functional connections between the precuneus subregion and the cerebellum (34). The cingulate gyrus is a key node of the default network and an important part of the limbic system, which has extensive connections with the hippocampus, frontotemporal cortex, etc. (35). Moreover, studies have confirmed the existence of hyperconnectivity between the cerebellum and the posterior cingulate gyrus (36). Studies have shown that damage to the posterior cingulate impairs episodic memory and retrieval, especially the spatial component (37). The middle cingulate gyrus is thought to regulate attention and executive function (38). The temporal cortex is also a member of the default network that is involved in various cognitive functions, including memory, auditory cognition, and semantics (39). Among them, the middle temporal gyrus is thought to play a role in language-related tasks such as lexical comprehension and semantic cognition (40), and the superior temporal gyrus is closely related to speech perception (41). Additionally, reduced efficiency in the regions of the lenticular nucleus and the putamen nucleus was also found. The lentiform nucleus and putamen are involved in motor coordination and language functions, and studies have shown that they have network connections with the cerebral cortex and cerebellum (42). We also found abnormal function of the angular gyrus, rectal gyrus and cuneiform in our study. The angular gyrus plays important roles in semantic processing, memory retrieval, attention and spatial cognition, reasoning, and social cognition (43). The cuneus is involved in regulating working memory (44). An animal experiment demonstrated functional connectivity between the mouse cerebellum and prefrontal cortex and showed that the prefrontal cortex mediates cerebellar-mediated social and repetitive/inflexible behaviors (45). Our findings support that cognitive impairment may be primarily attributable to reduced local connectivity efficiency in brain regions involved in the default network and the limbic system after cerebellar infarction. In addition, the changes in network connectivity of bilateral brain regions after cerebellar infarction may be related to the regulation of the cerebellum, but further studies are needed for verification.

At the same time, we conducted a correlation analysis between abnormal network characteristics and changes in cognitive scale scores. In the left cerebellar infarction group, Eg values were positively correlated with MMSE scores. In addition, Eg values and regional efficiency of the DCG.R were positively correlated with DST scores. In the right cerebellar infarction group, Lp values were negatively correlated with MMSE scores, and Eg values were positively correlated with MMSE and RAVLT-A scores and El values and regional efficiency of the PCUN.L were negatively correlated with TMT-B scores. Based on the above research results, the cerebellum may play an important role in cognitive function, especially in memory. At the same time, there was a significant correlation between attentional impairment associated with the left cerebellum and brain regions in the right hemisphere, and executive function associated with the right cerebellum was significantly correlated with brain regions in the left hemisphere. Combined with the results of brain region efficiency studies, we believe that the cerebellum has regulatory effects on both sides of the cerebrum but may be more closely related to the contralateral cerebrum. This is consistent with the perspective from previous research suggesting that “cerebrocerebellar loops” have the characteristics of cross-connection (46). This requires further research.

Our study provides a new perspective for further exploration of cerebellar cognitive impairment. It reveals that the cerebellum may have a regulatory and integrative role in cognitive function. In previous studies on schizophrenia and multiple sclerosis, they were considered to be a neural oscillatory connectomopathy (47, 48). At the same time, our study also found that extensive brain network connectivity disintegrates after cerebellar injury, so it can also be considered a connectomopathy. At present, the treatment of neural network destruction is a research hotspot, and neuromodulation is one of these important methods. By definition, neuromodulation is the change in neural activity that occurs by stimulating a specific area of the nervous system. The stimuli mentioned can be electrical, magnetic or chemical. This method is used in various diseases, such as transcranial magnetic stimulation for Alzheimer's disease (49). Furthermore, neuromodulation can also be achieved through the use of neural implants, which is applied nowadays, especially in Parkinson's disease, and the use of microchips and prostheses to treat various symptoms of different neurological disorders has received significant attention (50). There are extensive neural network connections between the cerebellum and the cerebrum, and currently, the treatment of dementia is still a worldwide problem. Using the cerebellum as a stimulation target to treat dementia through neuromodulation may become a new treatment method.

## Limitations

Although we obtained certain research results, this study has certain limitations. First, this study was a single-center study with a relatively small sample size, and the relevant results still need to be confirmed by more research and further expansion of the sample size. Second, Because of the small sample size, the study did not differentiate the subregions of the cerebellum. In future work, we will further study each subregion of the cerebellum. Thirdly, there have been studies using multimodal MRI, such as combined PET-CT and cerebrospinal fluid techniques, to improve the accuracy of imaging diagnostic assessments. Therefore, in future research, we also need to update the technology, such as using multimodal MRI examinations, to improve the accuracy of the research results.

## Conclusion

In conclusion, this study compared the brain network properties between patients with cerebellar infarctions on different sides and healthy subjects; we found that unilateral cerebellar infarction can cause disruptions in the bilateral cerebral hemispheres. The efficiency values in key brain regions were decreased, and the abnormal changes were closely related to the degree of cognitive impairment in the patients. These findings suggest that cognitive impairment after cerebellar infarction is associated with reduced connectivity and information transfer in certain brain regions, that the cerebellum may be involved in the integration and regulation of brain network connections. The results of this study provide a basis for understanding the underlying neural mechanisms of cerebellar cognitive impairment, and provide some guidance for clinical work.

## Data availability statement

The original contributions presented in the study are included in the article/supplementary material, further inquiries can be directed to the corresponding authors.

## Ethics statement

The studies involving human participants were reviewed and approved by the Ethics Committee of the Affiliated Brain Hospital of Nanjing Medical University. The patients/participants provided their written informed consent to participate in this study.

## Author contributions

JS designed the study and critically revised the content of the paper. DW performed clinical data collection, research, and manuscript writing. QY and XL helped with data analysis. JH assisted with magnetic resonance data acquisition and analysis. All authors contributed to the article and approved the submitted version.

## Conflict of interest

The authors declare that the research was conducted in the absence of any commercial or financial relationships that could be construed as a potential conflict of interest.

## Publisher's note

All claims expressed in this article are solely those of the authors and do not necessarily represent those of their affiliated organizations, or those of the publisher, the editors and the reviewers. Any product that may be evaluated in this article, or claim that may be made by its manufacturer, is not guaranteed or endorsed by the publisher.
